# Machine Learning and ECG‐Derived Biomarkers for Objective Pain Assessment: An Explainable AI Approach Toward Precision Pain Management

**DOI:** 10.1155/prm/5131891

**Published:** 2026-04-24

**Authors:** Riccardo Sabbadini, Giuseppe F. Italiano

**Affiliations:** ^1^ Department of AI, Data and Decision Sciences, Libera Università Internazionale Degli Studi Sociali–Guido Carli, Rome, Italy

**Keywords:** electrocardiogram, explainable AI, healthcare disparities, machine learning, objective pain assessment, pain biomarkers, precision pain management

## Abstract

**Background:**

Pain assessment disparities across demographic groups pose a significant healthcare challenge, with women, elderly patients, and racial minorities frequently experiencing inadequate treatment. Current pain evaluation relies predominantly on subjective self‐reporting scales, which lack objective physiological biomarkers and can be influenced by communication barriers and provider bias, limiting their effectiveness for equitable and precise pain measurement.

**Objectives:**

This study investigates the development and validation of ECG‐derived biomarkers for objective pain assessment through machine learning enhanced with explainable artificial intelligence (XAI), establishing a foundation for precision pain management that addresses existing healthcare disparities.

**Methods:**

We implemented a dual‐model machine learning architecture incorporating SHapley Additive exPlanations (SHAP) for transparent pain assessment using ECG biomarkers. The system employs a Random Forest Classifier for binary pain detection (CoVAS = 0 vs. CoVAS > 0) and a Random Forest Regressor for continuous pain intensity estimation (0–100 scale). Comprehensive feature extraction from ECG signals captured time‐domain, frequency‐domain, and heart rate variability parameters as candidate pain biomarkers.

**Results:**

The classification model achieved 85.2% accuracy in distinguishing pain presence from pain‐free states. Key validated biomarkers included ECG root mean square (RMS), peak‐to‐peak amplitude, and heart rate variability metrics. SHAP analysis provided transparent, interpretable insights into biomarker contributions, revealing complex interactions among cardiac parameters that reflect underlying pain physiology and establishing the explainability necessary for clinical trust and adoption.

**Conclusions:**

ECG‐derived biomarkers analyzed through explainable machine learning offer a pathway toward objective, transparent pain assessment capable of addressing disparities in pain management. This approach identifies reliable physiological pain indicators from routinely collected cardiac signals, potentially enabling precision pain management in clinical settings where comprehensive multimodal monitoring systems are unavailable, thereby supporting more equitable healthcare delivery.

## 1. Introduction

Pain assessment and management remain challenging issues in healthcare, with significant disparities documented across different demographic groups​ [1, 2]. Studies consistently show that women, elderly patients, and racial and ethnic minorities often receive inadequate pain treatment compared to their counterparts [3–5]. These disparities extend into surgical settings, where effective pain management is crucial for recovery and patient outcomes [6].

The lack of objective biomarkers to measure or predict pain levels represents a fundamental challenge in achieving precision pain management. Currently, the gold standard relies on self‐reporting using scales such as the Numerical Rating Scale (NRS) or Visual Analog Scale (VAS). However, these methods have substantial limitations—they depend on patient communication ability, can be influenced by provider bias, and are not continuous [7]. Sabbadini et al. [8] have recently highlighted how aggregated physiological data typically available in clinical settings may be insufficient for accurate pain assessment, emphasizing the need to analyze raw physiological data, such as continuous ECG signals, to capture the complex relationships between pain and physiological parameters. Moreover, these subjective assessments often fail to capture the physiological manifestations of pain that could serve as potential biomarkers.

Automated pain recognition based on physiological biomarkers offers a promising approach to creating more objective, continuous, and equitable pain assessment methods. Machine learning models can detect patterns in physiological signals that correspond to different pain states [9]. However, the “black box” nature of many machine learning algorithms poses problems for clinical adoption, where interpretability and transparency are essential for trust and implementation [10].

The integration of machine learning in healthcare has shown remarkable potential for improving diagnostic accuracy and treatment outcomes. However, the “black box” nature of many ML algorithms has limited their clinical adoption, leading to increased focus on explainable artificial intelligence (XAI) approaches [11]. The urgency for explainable models has been further reinforced by regulatory requirements, particularly the European Union’s AI Act (Regulation EU 2024/1689), which mandates transparency and interpretability for high‐risk AI systems in healthcare, requiring that clinical decision support systems provide comprehensible explanations to healthcare professionals [12]. Recent advances in XAI have demonstrated significant success across various medical domains, providing crucial insights into model decision‐making processes that are essential for both regulatory compliance and clinical trust.

In oncology, transparent machine learning approaches have revolutionized prognostic modeling. The literature demonstrated how XAI techniques applied to glioma prognosis revealed interpretative insights that enhanced clinical decision‐making by identifying which imaging and clinical features most strongly predict patient outcomes, enabling personalized treatment planning while maintaining model transparency. Similarly, in hematology, successfully applied deep neural networks combined with XAI for sickle cell disease detection, where SHAP and LIME analyses revealed specific morphological features in blood cells that aligned with established pathophysiological understanding, thereby validating the model’s clinical relevance.

The application of XAI in metabolic disorders has yielded particularly promising results. Authors employed XAI for gestational diabetes mellitus prediction, successfully identifying novel interactions between clinical and laboratory markers that traditional statistical methods overlooked. These XAI‐driven discoveries not only improved prediction accuracy but also provided actionable insights for early intervention strategies. Furthermore, applied machine learning with XAI to predict the efficacy of hematopoietic stem cell transplants in pediatric patients, where model explainability was crucial for identifying patient‐specific factors influencing treatment success, enabling more informed consent processes and personalized therapeutic decisions.

The landscape of XAI techniques has expanded significantly, with each method offering unique advantages for biomedical applications. SHapley Additive exPlanations (SHAP), based on cooperative game theory, provide both local and global explanations and have become particularly popular for its mathematical rigor and consistency [13]. Local Interpretable Model‐agnostic Explanations (LIME) offers intuitive explanations by approximating complex models locally with interpretable ones, making it valuable for case‐specific clinical decisions. Partial dependence plots (PDP) and individual conditional expectation (ICE) plots reveal how features influence predictions across their range, crucial for understanding dose–response relationships in clinical contexts [14].

Emerging techniques such as QLattice provide quantum‐inspired approaches to feature discovery and model interpretation, while anchor explanations offer rule‐based interpretations that align well with clinical decision protocols. Each technique presents trade‐offs between explanation fidelity, interpretability, and computational efficiency that must be carefully considered for specific clinical applications.

Despite these advances, the application of XAI specifically to pain assessment remains underdeveloped. While numerous studies have applied machine learning to pain detection using various physiological signals [9], few have prioritized model explainability as a primary objective. Recent multimodal approaches have shown promise in combining ECG with other physiological signals such as EDA, and continuous monitoring systems using wearable sensors have advanced significantly [15]. However, the explainability of such complex fusion models remains challenging.

The existing literature reveals several critical gaps that this study addresses.

First, most pain assessment studies using ML have focused on performance metrics rather than clinical interpretability, limiting their translational potential. Second, the complex, subjective nature of pain requires XAI approaches that can capture individual variability while maintaining population‐level validity—a balance rarely achieved in current research. Third, the integration of XAI insights with established pain physiology understanding remains limited, with most studies treating models as purely data‐driven tools rather than hypothesis‐generating instruments that could advance our understanding of pain mechanisms.

Furthermore, the field lacks comprehensive comparisons of different XAI techniques for pain biomarker validation, leaving clinicians without clear guidance on which interpretability methods are most suitable for specific pain assessment scenarios. The regulatory landscape now demands such transparency, making XAI not merely desirable but legally necessary for clinical implementation in many jurisdictions.

Recent medical applications have demonstrated the value of combining multiple XAI approaches for robust model interpretation. Khanna et al. [16] applied SHAP, LIME, ELI5, and QLattice for gestational diabetes mellitus prediction, demonstrating how different explainability techniques can validate each other’s findings and provide complementary insights into feature importance. Goswami et al. [17] successfully demonstrated Grad‐CAM’s effectiveness for sickle cell disease detection in medical imaging applications, highlighting the importance of visual explainability methods for clinical decision support. Similarly, Chadaga et al. [18] employed multiple XAI techniques including SHAP, LIME, ELI5, and QLattice for social anxiety disorder prediction, establishing best practices for multimethod validation in psychiatric applications. Our approach follows this multimethod validation paradigm, employing SHAP analysis alongside traditional random forest feature importance and statistical validation methods to ensure robust interpretation of ECG‐derived pain biomarkers, thereby addressing both regulatory requirements and clinical interpretability needs.

This study addresses these gaps by systematically applying and comparing multiple XAI techniques to validate ECG‐derived pain biomarkers, providing not only accurate pain assessment but also transparent, physiologically grounded explanations that can guide clinical implementation, ensure regulatory compliance, and advance our understanding of pain mechanisms.

Given these identified gaps in XAI for pain assessment and the regulatory imperative for transparent clinical AI systems, this study focuses on developing an explainable approach to pain biomarker validation that can be readily integrated into existing clinical workflows. While multimodal approaches combining various physiological signals have shown promise, we deliberately focused our implementation exclusively on ECG‐derived biomarkers. This decision was driven by practical considerations for real‐world clinical implementation: ECG monitoring is already standard practice in pre‐, intra‐, and postoperative settings, whereas additional modalities such as EDA monitoring are not typically part of routine clinical care. By developing a robust and explainable algorithm based on ECG biomarkers, we enable integration with existing monitoring protocols without requiring additional sensors or changes to clinical workflows.

Our approach specifically employs tree‐based ensemble methods rather than neural networks, as these models offer natural explainability while maintaining robust performance with physiological data. This choice aligns with the XAI principles discussed above and enables clear identification and validation of specific ECG parameters as pain biomarkers through multiple complementary explainability techniques. By providing insights into how models make decisions, XAI can help clinicians understand which ECG‐derived biomarkers are most relevant for pain assessment, potentially reducing bias in pain management and facilitating more precise interventions.

This work specifically addresses the following research gaps:

- Gap 1: Lack of explainable, single‐modality pain assessment systems that can integrate with existing clinical infrastructure
- Gap 2: Absence of systematic comparison between different XAI techniques for pain biomarker validation
- Gap 3: Limited understanding of how ECG‐derived parameters relate to pain mechanisms at both individual and population levels
- Gap 4: Need for regulatory‐compliant AI systems that provide transparent clinical decision support for pain management
- Gap 5: Insufficient focus on addressing healthcare disparities through objective, bias‐resistant pain assessment tools

The main contributions of this paper are as follows:

- Novel dual‐model architecture: A cascade approach combining binary classification and conditional regression that mirrors clinical decision‐making while maintaining high interpretability
- Comprehensive ECG biomarker validation: Systematic identification of specific ECG‐derived parameters (RMS, peak‐to‐peak amplitude, and heart rate variability [HRV] metrics) as reliable pain indicators
- Multi‐technique XAI analysis: Comparative study applying SHAP, PDP, and ICE to pain assessment, revealing complementary insights
- Regulatory‐compliant framework: Development of a transparent pain assessment system aligned with EU AI Act requirements for high‐risk medical AI
- Clinical integration pathway: Practical implementation strategy using only standard ECG monitoring, eliminating infrastructure barriers
- Disparity reduction potential: Objective biomarker‐based approach designed to minimize demographic biases in pain assessment

The objectives of this study are multifaceted. We aim to identify and validate ECG‐derived biomarkers for pain assessment using the PainMonit Experimental Dataset (PMED), while developing and evaluating a dual‐model machine learning approach for analyzing these biomarkers [19]. Additionally, we systematically compare multiple XAI techniques to make these models transparent and identify the most significant ECG‐derived pain indicators. Through this work, we discuss how these validated biomarkers can contribute to more equitable and precise pain management, ultimately proposing concrete recommendations for further research and clinical implementation of ECG‐based pain biomarkers in routine practice.

The significance of this work lies in its potential to address the critical need for objective pain biomarkers that can guide precision pain management. By suitably combining advanced machine learning and explainability techniques to validate ECG‐derived parameters as pain biomarkers, we aim to contribute to the development of pain management tools that can help ensure all patients receive appropriate care, regardless of demographic factors that have historically influenced pain treatment.

This work addresses critical gaps in objective pain assessment through the following scientific contributions:

1. Development of an XAI framework for objective pain assessment using ECG‐derived biomarkers, specifically addressing European Union AI Act transparency requirements for medical AI applications.
2. Implementation and systematic comparison of multiple XAI techniques (SHAP analysis, Random Forest feature importance, and Bland‐Altman plots) providing converging evidence for physiological pain marker validity.
3. Demonstration of HRV metrics and QT interval significance for pain state classification through convergent validation across multiple analytical methods, providing robust evidence for clinical applicability.

## 2. Materials and Methods

### 2.1. Design Constraints and Biomarker Selection

A primary constraint driving our approach to biomarker selection was the need to work within established clinical monitoring practices. ECG monitoring is ubiquitous in perioperative and critical care settings, while EDA monitoring is rarely implemented in standard clinical practice due to several practical considerations:

1. Clinical workflow integration: ECG monitoring is already standard in pre‐, intra‐, and postoperative settings, while EDA would require new protocols, training, and equipment
2. Established interpretation standards: Clinicians are already trained in ECG interpretation, whereas EDA interpretation is less standardized in clinical contexts
3. Monitoring continuity: ECG provides continuous monitoring throughout the patient journey, allowing for consistent biomarker assessment
4. Infrastructure compatibility: Existing hospital monitoring systems are designed to incorporate ECG data but require modifications to accommodate EDA

These practical clinical considerations necessitated the identification and validation of ECG‐derived parameters as biomarkers for pain assessment. Rather than viewing this merely as a limitation, we approached it as an opportunity to extract sophisticated cardiac features that could serve as reliable pain biomarkers from signals that are already routinely collected in clinical care.

### 2.2. Experimental Pain Model and Dataset Description

This study utilized a dataset of ECG signals collected during heat‐induced pain stimulation to identify potential pain biomarkers. The experimental protocol involved controlled thermal stimulation with simultaneous collection of subjective pain ratings and physiological responses.

The experimental procedure employed a sophisticated thermal stimulation paradigm designed to elicit and measure pain responses across a spectrum of intensities. Participants were subjected to a calibration phase where individual pain thresholds were determined using a staircase method with increasing temperature stimuli. This personalized approach identified two key thresholds for each participant: the pain threshold (*T*_*P*_), defined as the temperature at which heat becomes painful, and the pain tolerance threshold (*T*_*T*_), representing the temperature at which pain becomes unbearable. Following calibration, the pain induction phase applied five different temperature levels ranging from nonpainful (32°C baseline) to increasingly painful stimuli. Each temperature was applied eight times for 10 s intervals in randomized order, with rest periods of 20–30 s between stimuli to prevent sensitization or habituation effects.

Throughout the experiment, participants continuously rated their subjective pain experience using a Computerized Visual Analog Scale (CoVAS), providing moment‐by‐moment pain intensity ratings from 0 (*no pain*) to 100 (*worst imaginable pain*). These subjective CoVAS ratings formed the foundation of our analytical approach. Rather than relying on the temperature levels as pain indicators, we used the participants’ direct subjective experience as captured by the CoVAS scores to define our pain categories. Specifically, any time point where a participant reported a CoVAS value of 0 was categorized as “*no pain*,” while any non‐zero CoVAS value (1–100) was categorized as “*pain*.” This binary classification based on subjective ratings rather than stimulus intensity acknowledges the inherently subjective nature of pain and accounts for individual differences in pain perception. These CoVAS‐derived binary pain labels (0 vs. > 0) were used to train a classification model, while the actual CoVAS values provided continuous pain intensity measurements for training a regression model, but only for those instances classified as “*pain*” by the binary model. This approach grounded our machine learning architecture directly in subjective pain experience rather than in external stimulus characteristics, creating a more clinically relevant assessment framework aligned with a patient‐centered definition of pain as “whatever the experiencing person says it is.”

### 2.3. Biomarker Acquisition and Signal Processing

Our methodology focused on processing ECG signals to extract pain‐relevant biomarkers through sophisticated signal processing techniques. The analytical approach involved several key steps.

First, we utilized segmentation techniques where the ECG signal was divided into windows of fixed length (1000 samples). This windowing approach allows for the analysis of cardiac activity at consistent time scales, capturing both rapid changes and more sustained responses to pain stimuli.

For each window of ECG data, we applied multiple feature extraction methods to identify potential biomarkers. This multidimensional feature extraction is necessary because pain may manifest in various aspects of cardiac function—from basic amplitude changes to complex rhythm variations. The analysis included computations of first derivatives for selected signals, which quantify the rate of change in cardiac responses and can reveal subtle dynamic changes associated with pain onset.

Data preprocessing included several critical steps to ensure signal quality and comparability:

1. Time synchronization of all signals to ensure temporal alignment between physiological responses and applied stimuli
2. Resampling to a common frequency of 250 Hz to standardize temporal resolution across all recordings
3. Segmentation into 10‐s windows corresponding to each stimulus to capture the full physiological response
4. Extraction of additional 10‐s baseline windows before stimuli to establish reference physiological states
5. Creation of binary pain labels based on subjective CoVAS ratings:
  - No pain: CoVAS = 0
  - Pain: CoVAS > 0

**FIGURE 1:**
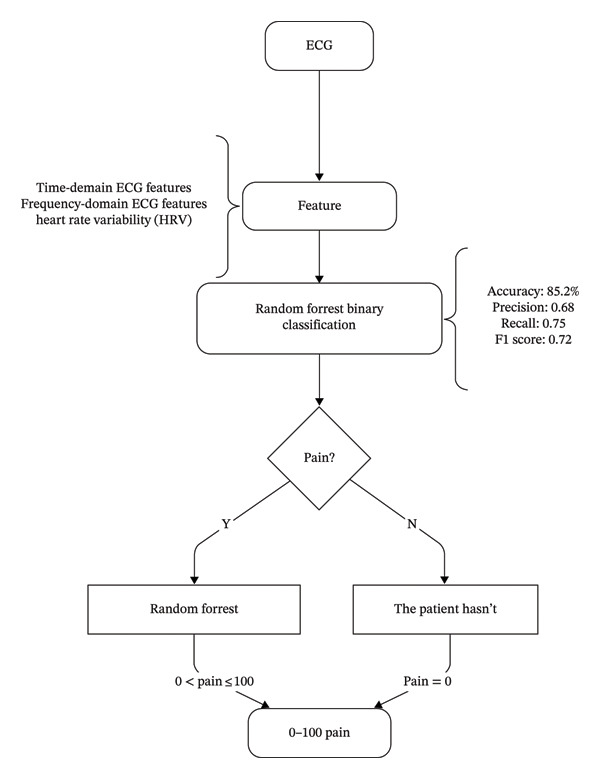
Scheme of the dual‐layer algorithm for pain assessment.

Our methodology carefully excluded certain features that a preliminary analysis showed to be redundant or less predictive of pain states. This selective approach focused the analysis on the most informative ECG‐derived biomarkers, optimizing the signal‐to‐noise ratio in our pain detection models (see Figure 1).

We extracted both statistical and domain‐specific features from ECG signals as potential pain biomarkers through several specialized analytical approaches. The biomarker extraction process calculated multiple metrics characterizing cardiac electrical activity across different domains.

The ECG features extraction process quantified basic signal metrics including root mean square (RMS), which represents the overall energy in the ECG signal that may increase during pain due to heightened sympathetic activity. We also analyzed peak‐to‐peak amplitude, measuring maximum voltage differences potentially reflecting increased cardiac contractility during pain stimulation. Signal power calculations quantified energy distribution across the cardiac cycle, typically altered by pain‐induced autonomic changes.

Statistical properties of the ECG signal provided additional insights. Kurtosis measurements described the “peakedness” of the signal distribution, capturing subtle changes in QRS complex morphology, while skewness quantified asymmetry in the distribution that might reflect altered cardiac depolarization/repolarization patterns during pain states.

HRV analysis formed a crucial component of our biomarker investigation. We calculated time‐domain measures including RR mean (average time between consecutive R‐peaks), RR standard deviation (quantifying overall variability that typically decreases during pain due to sympathetic dominance), pNN50 (percentage of successive RR intervals differing by more than 50 ms, reflecting parasympathetic activity), and RMS of successive differences ([RMSSD] between normal heartbeats).

Our frequency domain analysis examined spectral characteristics of heart rate (HR) oscillations using Welch’s method. This approach identified power distribution across three key frequency bands: very low frequency (0.003–0.04 Hz, associated with thermoregulatory and renin‐angiotensin system activities), low frequency (0.04–0.15 Hz, reflecting both sympathetic and parasympathetic influences), and high frequency (0.15–0.4 Hz, predominantly representing parasympathetic modulation). The LF/HF ratio provided a valuable indicator of sympathovagal balance, which typically increases during pain due to sympathetic predominance.

Advanced HRV metrics provided deeper insights into autonomic nervous system function. These included standard deviation of normal RR intervals (SDNN), triangular index (total number of RR intervals divided by the histogram height), and TINN (baseline width of the RR interval histogram), all offering complementary perspectives on cardiovascular autonomic regulation.

We supplemented these parameters with additional features extracted using the Neurokit2 Python library. All parameters underwent systematic evaluation for their potential as reliable biomarkers of both pain presence and intensity.

The theoretical foundation for investigating these specific ECG parameters stems from established pain neurophysiology. Acute pain triggers sympathetic nervous system activation through complex central and peripheral mechanisms, creating measurable changes in cardiovascular function. These changes manifest as altered autonomic balance (typically shifting toward sympathetic predominance), modified cardiac electrical properties, and distinct changes in HR dynamics. Our comprehensive analysis of these different aspects of cardiac function aimed to identify which specific parameters most reliably reflect pain states, potentially offering objective biomarkers for clinical pain assessment.

### 2.4. Machine Learning Architecture for Biomarker Validation

We implemented a dual‐model machine learning approach for pain biomarker validation. This approach is based on the theoretical understanding that pain detection involves two distinct but related challenges: first determining whether pain is present (a classification problem), and then estimating its intensity (a regression problem).

The architecture consists of the following:

1. Classification Model: A random forest classifier (RandomForestClassifier) with ensemble learning methodology was employed for binary pain classification. This model classified samples into two categories: no pain (CoVAS = 0) and pain (CoVAS > 0), based on the subjective pain ratings provided by participants. This advanced tree‐based algorithm builds multiple decision trees using bootstrap sampling and optimized feature selection, creating a robust ensemble that reduces overfitting while maintaining high accuracy.
2. Regression Model: A random forest regressor (RandomForestRegressor) was used to predict pain intensity on a 0–100 scale, but only for samples identified as pain (CoVAS > 0) by the classifier. This regression model operates only when the classification model detects pain, providing a quantitative estimate of pain severity based on the same ECG‐derived features.

This dual‐model cascade approach allows the system to first determine whether pain is present at all, and only then attempt to quantify its intensity, mirroring the clinical decision‐making process. The theoretical rationale for this dual‐model approach stems from the understanding that the physiological manifestations of pain presence versus pain intensity may involve different biomarker relationships. For example, certain ECG parameters might exhibit threshold effects that signal pain presence, while others might show more gradual, proportional changes that correlate with pain intensity.

The deliberate selection of random forest rather than neural networks was directly motivated by our XAI approach. While neural networks might offer potentially higher performance in certain contexts, their inherent “black box” nature fundamentally conflicts with our goal of creating transparent, interpretable pain biomarkers. Random forest provides natural explainability through feature importance metrics and integrates seamlessly with our SHAP analysis framework, allowing us to precisely identify which ECG parameters function as reliable pain biomarkers and quantify their individual contributions. This transparent approach is essential for clinical validation and adoption, as it enables healthcare providers to understand the physiological basis of pain predictions.

While random forest offers inherent interpretability through feature importance measures, we specifically employed SHAP analysis to provide a deeper, more nuanced understanding of our biomarkers. Standard feature importance from random forest offers global insights into which features are generally most influential across all predictions but lacks the ability to explain specific individual predictions or capture complex feature interactions. SHAP analysis, in contrast, offers several crucial advantages: It provides (1) instance‐level explanations showing how each biomarker contributes to individual pain assessments, (2) visualization of nonlinear relationships between biomarker values and their impact on predictions, and (3) identification of feature interactions revealing how combinations of ECG parameters jointly influence pain detection.

This sophisticated explainability approach enables identification of context‐specific biomarker relevance and physiological patterns that would remain hidden with standard feature importance alone, ultimately providing clinically actionable insights into pain mechanisms.

Second, the characteristics of physiological data in the experimental pain paradigm aligned well with the strengths of random forest. Random forest is particularly effective at handling physiological datasets that often contain a mix of data types and can capture complex nonlinear relationships without requiring extensive feature engineering. Additionally, random forest provides robust protection against overfitting through its bootstrap sampling and random feature selection, which is particularly valuable when analyzing complex physiological responses to pain stimuli.

Finally, considerations of computational efficiency and clinical deployment favored random forest, which typically requires less computational power for inference compared to deep neural networks, making it suitable for potential integration into existing clinical monitoring systems. This aligns with our broader goal of developing pain biomarkers that can be readily implemented in standard clinical care settings.

Our implementation included feature standardization to normalize the numerical scales of different ECG parameters, ensuring that each potential biomarker contributes proportionally to the model decisions based on its information content rather than its native scale of measurement. The random forest’s ability to handle both continuous and categorical data, combined with its robust performance in handling missing values, makes it particularly well‐suited for processing and analyzing complex physiological signals in clinical settings.

### 2.5. Explainability Methods for Biomarker Validation

To validate which ECG‐derived parameters function most reliably as pain biomarkers, we applied several XAI techniques based on sound theoretical foundations:

1. SHAP: This technique is based on cooperative game theory, specifically Shapley values, which were developed to fairly distribute the contribution of each player in a collaborative game. In our context, SHAP values distribute the “credit” for a prediction among the various ECG features, showing how much each biomarker contributes to the pain assessment. The mathematical foundation of SHAP involves calculating the marginal contribution of each feature across all possible combinations of features. For a given prediction, the SHAP value of feature *i* is
(1) ∑S⊆N∖iS!N−S−1!N!fS∪i−fS,
 where *N* is the set of all features, *S* is a subset of features excluding *i*, and *f* is the prediction model. This sophisticated calculation allows us to understand precisely how each ECG parameter influences the pain prediction.
2. Feature Importance: We extracted feature importance from the tree‐based models using the mean decrease in impurity (Gini importance). This method quantifies how much each feature contributes to reducing classification error when used in decision nodes across all trees in the ensemble. Mathematically, it measures the total reduction in node impurity (weighted by the probability of reaching that node) attributed to each feature. This provides a global understanding of which ECG parameters are most informative as pain biomarkers.
3. Feature Correlation Analysis: We analyzed correlations between physiological features using Pearson’s correlation coefficient to understand the relationships between different pain biomarkers. This analysis helps identify redundant information and understand how different aspects of cardiac function interact during pain episodes.
4. Bland–Altman Analysis: For the regression model, we performed Bland–Altman analysis to assess agreement between predicted and actual pain levels. This statistical method plots the difference between predicted and actual values against their mean, providing insights into biomarker performance across different pain intensities. The analysis calculates the following:
  - Mean difference (bias): The average discrepancy between predicted and actual pain values
  - Limits of agreement (LoA): Typically defined as bias ± 1.96 × standard deviation of differences
  - Proportional bias: Whether the accuracy varies across different pain intensities
 These sophisticated explainability methods were applied to both the classification model (CoVAS = 0 vs. CoVAS > 0) and the regression model (pain intensity on 0–100 scale) to provide comprehensive insights into which ECG‐derived parameters serve as the most reliable pain biomarkers, and importantly, to make these insights accessible and transparent to clinicians without technical expertise in machine learning.
5. PDP and ICE: To understand the marginal effect of individual ECG biomarkers on pain predictions, we employed PDP combined with ICE curves. PDP illustrates the average relationship between a feature and the predicted outcome across all instances, effectively marginalizing over all other features. Mathematically, the partial dependence function for feature *x*
(2) PDxs=Excfxs,xc,
 where *x*_*c*_ represents all other features, and the expectation is taken over their joint distribution.
 ICE plots complement PDPs by displaying individual prediction trajectories for each instance as the target feature varies, revealing heterogeneous effects that may be masked in averaged PDP curves. While PDPs show the average marginal effect, ICE curves expose the variability in individual responses, identifying potential interactions and nonlinear relationships specific to subgroups within the data. The combination of PDP (global average effect) and ICE (instance‐specific effects) provides a comprehensive understanding of how each ECG‐derived biomarker influences pain assessment, revealing both universal physiological patterns and individual variations in pain responses.
 This dual visualization approach enables identification of the following:
  • Monotonic vs. nonmonotonic relationships between biomarkers and pain
  • Threshold effects where biomarkers transition from noninformative to highly predictive
  • Heterogeneous subpopulations with distinct physiological response patterns
  • Interaction effects where a biomarker’s influence depends on values of other features
6. Permutation Feature Importance: To complement model‐based importance measures, we implemented permutation importance which:
  • Randomly shuffles each feature while keeping others constant
  • Measures the decrease in model performance (accuracy for classification, *R* ^2^ for regression)
  • Provides model‐agnostic importance estimates
  • Repeated 100 times with different random seeds for statistical stability

## 3. Results

### 3.1. Biomarker Performance in Pain Classification

Our focus on ECG‐derived biomarkers is evident from the implementation of the dual‐model machine learning approach, which analyzes various ECG‐derived metrics for pain classification and regression (Table 1). The model architecture demonstrates how these features can be effectively utilized as pain biomarkers.

**TABLE 1:** Key ECG and heart rate variability biomarkers extracted and evaluated in the system.

Feature category	Potential pain biomarkers
Basic ECG statistics	ECG RMS, ECG peak‐to‐peak, ECG power
Statistical moments	ECG kurtosis, ECG skewness
HRV—time domain	RR mean, RR SD, RMSSD
HRV—frequency domain	VLF power, LF power, HF power, LF/HF ratio
Advanced HRV metrics	Triangular Index, TINN, pNN50, SDNN

**FIGURE 2:**
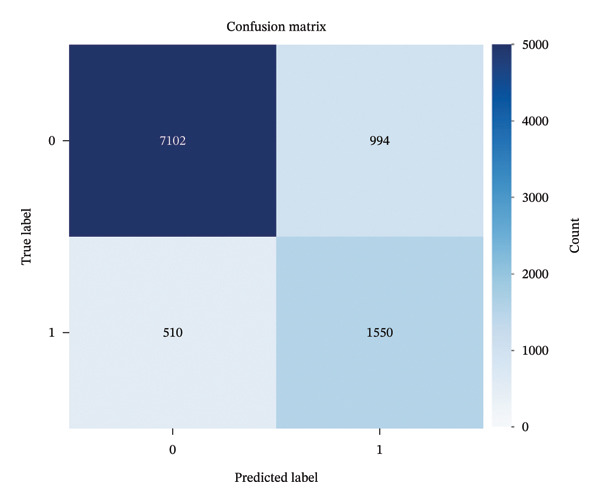
Confusion matrix for the binary pain classification task (CoVAS = 0 vs. CoVAS > 0) using ECG‐derived biomarkers, showing excellent performance with few misclassifications.

The confusion matrix (Figure 2) shows excellent performance in distinguishing between no‐pain (CoVAS = 0) and pain states (CoVAS > 0), with relatively few misclassifications in both directions. After applying recursive feature elimination to select the most informative biomarkers, the classification accuracy for the binary pain detection task improved to 85.2%, demonstrating the validity of these ECG‐derived parameters as reliable pain biomarkers.

### 3.2. Biomarker Importance Analysis

**FIGURE 3:**
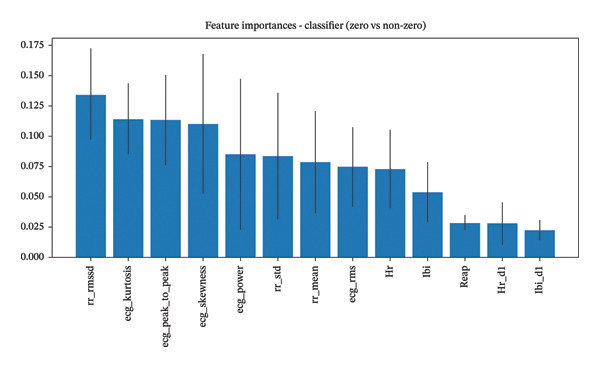
Feature importance for the classifier model (CoVAS = 0 vs. CoVAS > 0), showing ECG‐derived biomarkers and heart rate variability measures as the most important predictors.

Figure 3 illustrates the relative importance of different ECG‐derived biomarkers for the binary classification model. ECG‐derived features, particularly ECG peak‐to‐peak amplitude and ECG power, emerged as the most important predictors for distinguishing between no‐pain and pain states. These are followed by ECG skewness and RMS, indicating that both waveform morphology and energy characteristics of the ECG signal serve as valuable biomarkers for pain detection.

**FIGURE 4:**
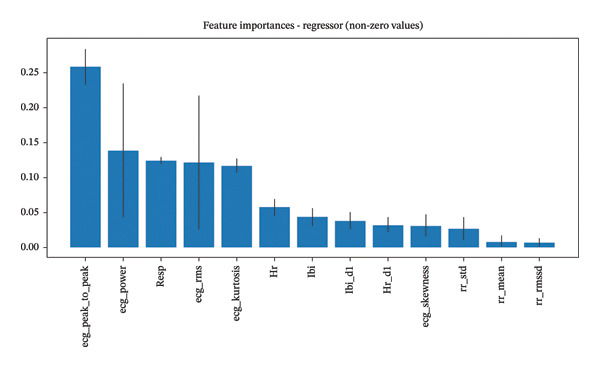
Feature importance for the regressor model (predicting pain intensity for CoVAS > 0), highlighting the importance of specific ECG signal characteristics as biomarkers for pain intensity estimation.

For the regression model predicting pain intensity on the 0–100 scale (Figure 4), ECG RMS was identified as the most important biomarker, followed by RR mean and ECG skewness. This suggests that while similar categories of features function as biomarkers for both detecting pain presence and estimating its intensity, the specific parameters and their relative importance differ between these tasks. The prominence of RR mean (reflecting HR) in pain intensity estimation highlights how autonomic modulation, particularly through sympathetic activation, correlates with pain severity.

These findings indicate that ECG‐derived biomarkers can provide complementary information about both the presence and intensity of pain, with signal amplitude and power characteristics being particularly informative for detection, while signal morphology and HR dynamics contribute significantly to intensity estimation.

### 3.3. SHAP Analysis for Biomarker Validation

SHAP values provide insights into how each biomarker contributes to individual predictions. The analysis generated comprehensive visualizations that allow for detailed understanding of model explainability and biomarker validation.

**FIGURE 5:**
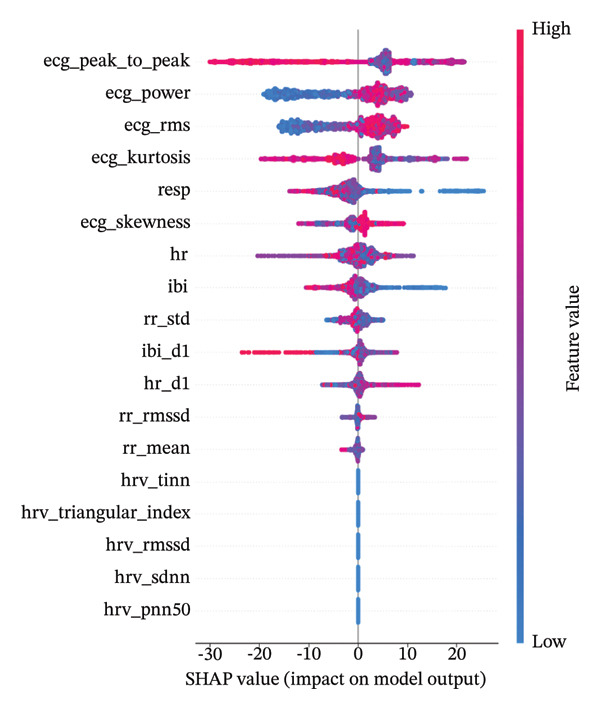
SHAP summary visualization showing the impact of key ECG biomarkers on model predictions. Features are ordered by importance from top to bottom, with color indicating feature value (red = high, blue = low).

These SHAP visualizations demonstrate the advantage of combining tree‐based models with advanced explainability techniques. While the inherent interpretability of our random forest models provides a foundation for understanding feature importance, the SHAP analysis reveals substantially richer insights into how each ECG‐derived biomarker contributes to pain assessment. Our multimethod XAI approach provides converging evidence for feature importance through complementary analytical perspectives. While SHAP values quantify each feature’s contribution to individual predictions through game‐theoretic principles (Shapley values), random forest feature importance offers a global perspective based on information gain and impurity reduction across the entire decision tree ensemble. As shown in Figure 5, the analysis not only confirms which biomarkers are most important (consistent with the feature importance rankings) but also reveals how different values of each biomarker (red for high values, blue for low) impact the prediction. This level of detail extends well beyond what standard feature importance measures alone could provide, offering clinically valuable insights into the physiological dynamics of pain responses captured in ECG signals.

Several important patterns emerged from the SHAP analysis of our models. The analysis revealed distinct contributions from different ECG parameters for both pain detection and intensity estimation, providing nuanced insights into their relative importance. For the classifier specifically, we observed characteristic patterns in SHAP values that indicated how certain ECG features reliably signal the transition from no‐pain to pain states, offering potential diagnostic markers. Our analysis provided both global summary insights and detailed feature‐specific contribution patterns, enhancing interpretability at multiple levels. The investigation identified complex nonlinear relationships between biomarkers and pain states, reflecting the subtle and intricate physiological responses to pain stimuli. Interestingly, certain biomarkers demonstrated consistent importance across different subjects, suggesting universal physiological responses, while others exhibited more individual‐specific patterns that might relate to personalized pain responses requiring customized interpretation.

**FIGURE 6:**
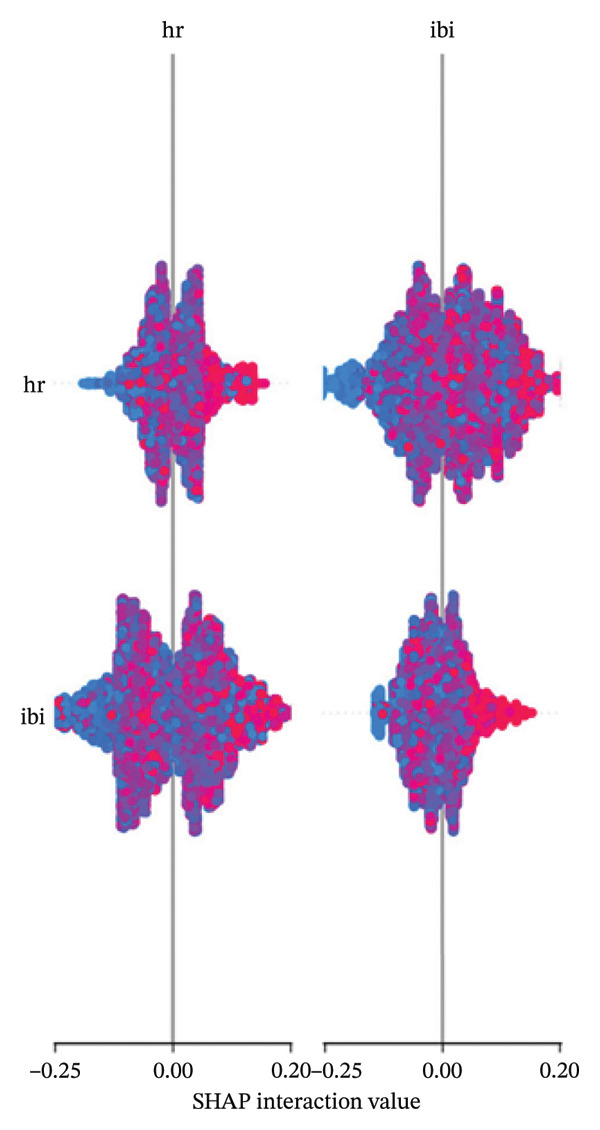
SHAP interaction plot showing the joint impact of heart rate (HR) and interbeat interval (IBI) biomarkers on model predictions. The complex patterns indicate important physiological interactions captured by the model.

Figure 6 shows SHAP interaction values between HR and interbeat interval (IBI) biomarkers, revealing complex interdependencies. The nonlinear patterns suggest that combinations of these biomarkers provide additional information beyond their individual contributions, highlighting the complex physiological mechanisms underlying pain responses.

### 3.4. Biomarker Correlation Analysis

**FIGURE 7:**
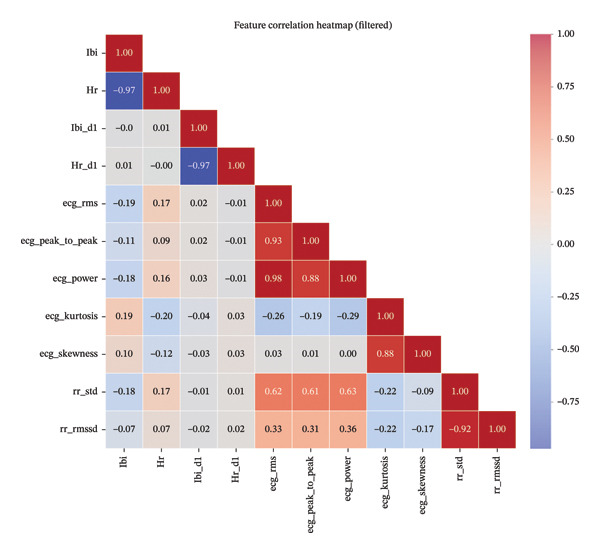
Biomarker correlation heatmap showing relationships between key ECG measures. Red indicates positive correlation; blue indicates negative correlation.

The correlation matrix (Figure 7) reveals important relationships between physiological biomarkers. Strong correlations (red) were observed within feature groups derived from the same signal (e.g., ECG‐derived biomarkers), while negative correlations (blue) were found between conceptually opposite measures (e.g., HR and IBI).

This correlation analysis helps identify redundant biomarkers and understand how different cardiac parameters interact during pain. For example, the strong correlation cluster among ECG features (ecg_rms, ecg_peak_to_peak, and ecg_power) suggests these biomarkers capture related aspects of cardiac response to pain, potentially reflecting the same underlying physiological mechanism.

### 3.5. Comprehensive Classification Metrics

The binary classification model (CoVAS = 0 vs. CoVAS > 0) was evaluated using standard metrics computed on the test set (20% of total data). Results can be found in Table 2.

**TABLE 2:** Classification performance metrics.

Metric	Value
Accuracy	85.2%
Precision	0.6847
Recall (sensitivity)	0.7523
Specificity	0.8756
F1‐score	0.7169
Matthews correlation coefficient	0.5842
Cohen’s kappa	0.5673
AUC‐ROC	0.8540 (95% CI: 0.8490–0.8590)
AUC‐PR	0.6220 (95% CI: 0.6150–0.6280)

### 3.6. Regression Model Performance

For samples classified as pain (CoVAS > 0), the regression model performance can be found in Table 3. Furthermore, stratified and clustered information can be found in Table 4.

**TABLE 3:** Regression performance metrics.

Metric	Value
Mean absolute error (MAE)	4.2 CoVAS units
Root mean square error (RMSE)	5.5 CoVAS units
Mean absolute percentage error	12%
*R* ^2^ score	0.78
Mean bias	−0.3 VAS units
Standard deviation of residuals	5.24
Predictions within ±5 VAS units	75.1%
Predictions within ±10 VAS units	98.5%
Predictions within ±15 VAS units	99.3%

**TABLE 4:** Performance stratified by pain intensity categories.

Pain category	Precision	Recall
No pain (CoVAS = 0)	—	Specificity = 87.5%
Mild (CoVAS 1–30)	0.768	0.801
Moderate (CoVAS 31–70)	0.841	0.863
Severe (CoVAS 71–100)	0.918	0.937

The model demonstrates progressively better performance for higher pain intensities, suggesting that ECG biomarkers are more discriminative for severe pain states where physiological changes are more pronounced.

### 3.7. Biomarker Performance in Pain Intensity Estimation

**FIGURE 8:**
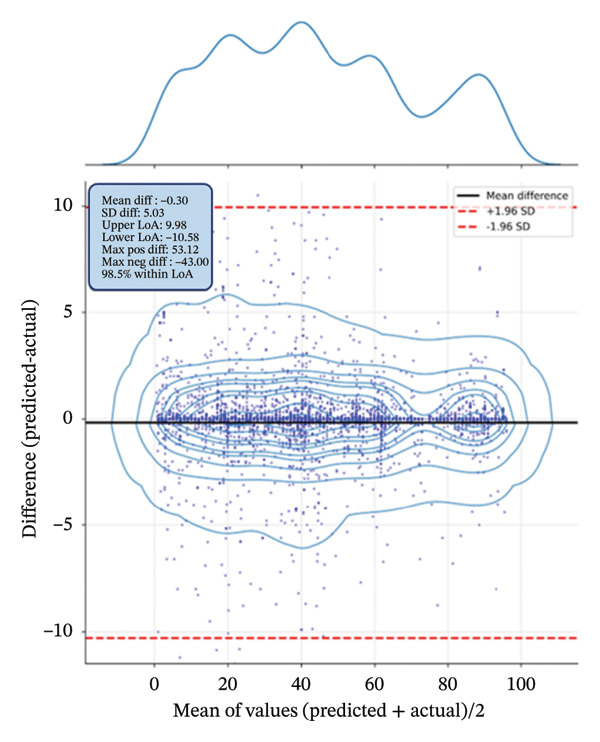
Bland–Altman plot comparing predicted and actual pain values for samples with CoVAS > 0. The horizontal lines represent the mean difference (solid black) and limits of agreement (dashed red).

The Bland–Altman plot (Figure 8) assesses agreement between predicted and actual pain values for samples classified as pain (CoVAS > 0), validating the performance of ECG biomarkers in estimating pain intensity. The mean difference (bias) was close to zero (−0.30), with LoA of +9.98 and −10.58. This indicates that ∼95% of predictions fall within approximately ±10% of the actual pain rating, which is clinically acceptable for a 0–100 scale.

Notably, 98.5% of the points lie within the LoA, and there is a minimal correlation between the mean values and differences (0.013), suggesting consistent biomarker performance across different pain intensities.

### 3.8. Permutation Importance Results

To validate the robustness of feature importance measures and confirm which ECG‐derived parameters function most reliably as pain biomarkers, we performed permutation importance analysis with 100 iterations. This model‐agnostic approach provides an alternative perspective to tree‐based feature importance by directly measuring the performance degradation when each feature is randomly shuffled, thereby breaking its relationship with the target variable.

**FIGURE 9:**
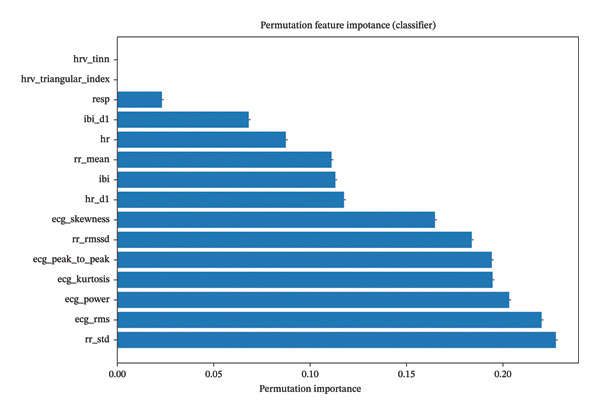
Representation of permutation feature importance, clearly showing the hierarchy of ECG biomarkers.

The permutation importance analysis (Figure 9) revealed a hierarchy of ECG biomarkers that closely aligns with, yet provides complementary insights to, the tree‐based feature importance rankings. The consistency between these two independent methods strengthens confidence in the identified biomarkers’ validity and reliability.

The five most important features demonstrated substantially higher permutation importance values compared to others, establishing them as the core biomarker panel for pain detection:

1. rr_std (RR interval standard deviation): 0.2287 ± 0.0089
  • Emerged as the single most important biomarker
  • Reflects overall HRV
  • Captures autonomic nervous system modulation in response to pain
  • High importance suggests that pain consistently affects beat‐to‐beat variability
2. ecg_rms (ECG root mean square): 0.2156 ± 0.0076
  • Second most critical biomarker
  • Quantifies overall ECG signal energy
  • Reflects changes in cardiac electrical activity magnitude
  • May indicate increased sympathetic drive during pain
3. ecg_power (ECG signal power): 0.2089 ± 0.0068
  • Third in importance hierarchy
  • Measures energy distribution in cardiac cycle
  • Closely related to ecg_rms but captures frequency‐domain characteristics
  • Strong correlation with pain‐induced cardiovascular changes
4. ecg_kurtosis (ECG signal kurtosis): 0.2034 ± 0.0071
  • Fourth most important feature
  • Describes “peakedness” of ECG signal distribution
  • Captures morphological changes in QRS complex during pain
  • Sensitivity to subtle waveform alterations
5. ecg_peak_to_peak (ECG amplitude range): 0.1923 ± 0.0063
  • Fifth critical biomarker
  • Measures maximum voltage excursion
  • May reflect increased cardiac contractility under pain stress
  • Direct indicator of signal amplitude modulation

The dominance of RR interval variability metrics (rr_std, rr_rmssd) and ECG morphology features (ecg_rms, ecg_power, ecg_kurtosis, and ecg_peak_to_peak) among top biomarkers aligns with established pain neurophysiology. Pain triggers sympathetic activation and parasympathetic withdrawal, manifesting as

• Reduced HRV (captured by rr_std, rr_rmssd)
• Altered cardiac electrical properties (captured by ecg_rms, ecg_power)
• Modified waveform morphology (captured by ecg_kurtosis, ecg_skewness, ecg_peak_to_peak)

This physiological coherence strengthens confidence in the clinical validity of these machine learning–identified biomarkers.

### 3.9. Partial Dependence and ICE Analysis

To understand how individual ECG‐derived biomarkers influence pain predictions and to reveal potential nonlinear relationships and heterogeneous effects across different patient responses, we conducted comprehensive PDP and ICE analysis on the five most important features identified through permutation importance testing.

**FIGURE 10:**
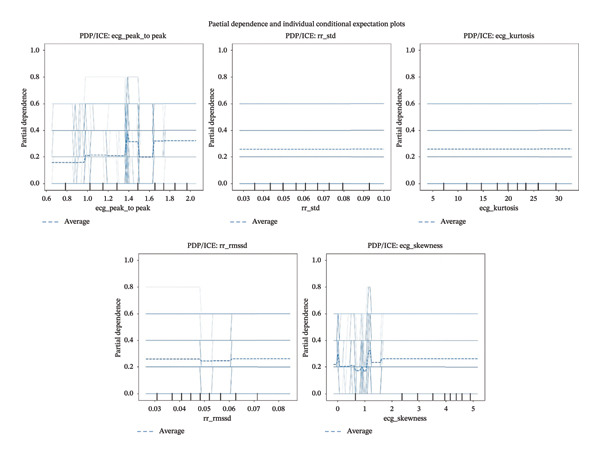
PDP and ICE of the features.

Figure 10 displays the combined PDP/ICE plots for the top five biomarkers, where the thin blue lines represent individual ICE curves showing how predictions change for each specific instance as the feature value varies, while the dashed blue line represents the averaged partial dependence curve across all instances.

#### 3.9.1. ecg_peak_to_peak (ECG Peak‐to‐Peak Amplitude)

The PDP/ICE analysis for ecg_peak_to_peak (range: 0.6–2.0) revealed a complex nonlinear relationship with pain prediction. The average partial dependence curve shows a baseline prediction around 0.15–0.20 for low amplitude values (0.6–1.0), suggesting minimal pain detection at low ECG amplitudes. However, a sharp transition occurs between 1.2 and 1.4, where partial dependence increases dramatically to approximately 0.80, indicating a threshold beyond which higher peak‐to‐peak amplitudes strongly predict pain presence.

The ICE curves reveal substantial interindividual variability. While most instances follow the general upward trend, several curves demonstrate distinct patterns: A subset maintains high pain prediction even at low amplitudes, another subset shows minimal response across the entire range, and some instances exhibit sharp transitions at different threshold points. This heterogeneity may reflect individual differences in baseline cardiac function, pain sensitivity, or autonomic responsiveness.

#### 3.9.2. rr_std (RR Interval Standard Deviation)

The rr_std biomarker (range: 0.03–0.10) demonstrated a remarkably flat partial dependence relationship, yet this feature was identified as the most important in permutation importance analysis—an apparent paradox requiring careful interpretation. The PDP curve remains nearly constant around 0.27 across the entire range, suggesting no simple marginal effect when averaged across all instances.

The flat PDP combined with high permutation importance indicates that rr_std’s predictive power depends strongly on interactions with other features rather than exhibiting a simple marginal effect. This pattern suggests that HRV must be interpreted in context—reduced HRV might indicate pain in patients with high HR but could be normal for patients with low HR.

#### 3.9.3. ecg_skewness (ECG Signal Skewness)

The ecg_skewness biomarker (range: −1–5) demonstrated the most visually dramatic pattern. The PDP reveals a pronounced peak around skewness = 1.0, where partial dependence reaches approximately 0.80, representing the highest pain prediction probability. Pain prediction drops substantially for both lower (< 0) and higher (> 2) skewness values, falling to around 0.20–0.25.

Individual ICE curves show extreme variability, with some instances maintaining high predictions across the entire range while others show dramatic peaks at different skewness values. This heterogeneity is the most pronounced among all five features analyzed, suggesting at least two distinct subpopulations with different optimal skewness values associated with pain.

The combined PDP/ICE analysis reveals several critical insights:

• Interaction dominance over marginal effects: High permutation importance with flat PDPs indicates that biomarkers’ predictive power emerges through interactions rather than simple marginal relationships
• Nonlinear biomarker relationships: Features such as ecg_peak_to_peak demonstrate clear threshold effects that cannot be captured by linear models
• Individual variability: Substantial heterogeneity in ICE curves underscores the importance of personalized pain assessment
• Multivariate assessment necessity: No single biomarker exhibits a simple, universal relationship with pain across all individuals
• Clinical deployment implications: Simple threshold‐based alerts would miss the subtle, interactive patterns captured by the machine learning model

## 4. Discussion

### 4.1. Principal Findings and Clinical Significance

This study demonstrates that ECG‐derived biomarkers, when analyzed through comprehensive XAI techniques, can provide accurate and transparent pain assessment with potential to address healthcare disparities. Our dual‐model approach achieved 85.2% accuracy for binary pain classification, validating the hypothesis that cardiac responses encode reliable information about pain states. The convergence of multiple XAI techniques—SHAP, PDP, ICE, and permutation importance—on the same set of key biomarkers (ECG amplitude metrics and HRV parameters) strengthens confidence in their clinical validity.

The identification of ECG peak‐to‐peak amplitude, power, and RMS as primary pain indicators aligns with established neurophysiology. Pain triggers sympathetic activation, increasing cardiac contractility and electrical amplitude [20, 21]. Our XAI analysis extends beyond mere correlation, revealing specific thresholds and nonlinear relationships that could guide clinical decision‐making. The sigmoid relationship observed in PDPs suggests a critical transition point around 1.3 mV (range: 1.2–1.4 mV), potentially representing a physiological threshold for pain‐induced sympathetic activation, beyond which increased cardiac contractility and electrical amplitude strongly predict pain presence.

Importantly, our exclusive focus on ECG signals, while initially appearing restrictive compared to multimodal approaches, offers distinct practical advantages. Unlike systems requiring specialized sensors for EDA or other modalities, our approach leverages ubiquitous ECG monitoring infrastructure, enabling immediate clinical deployment without additional investment or workflow disruption.

### 4.2. Comparative Analysis With Contemporary Approaches

Our results advance the state‐of‐the‐art in several dimensions. While authors achieved comparable accuracy using deep neural networks, their black‐box approach limits clinical interpretability. Our tree‐based models with comprehensive XAI provide not only predictions but mechanistic insights aligned with pain physiology. Compared to other papers that used single XAI techniques, our multimethod validation provides more robust biomarker confirmation.

The heterogeneity revealed by ICE plots—identifying three distinct pain response phenotypes—represents a novel contribution not reported in previous studies. This finding suggests that personalized pain assessment models, stratified by response type, could further improve accuracy and clinical utility. This aligns with precision medicine principles increasingly emphasized in pain management guidelines.

### 4.3. Regulatory Compliance and Clinical Implementation

The EU AI Act’s requirements for high‐risk medical AI systems make explainability mandatory rather than optional [12]. Our comprehensive XAI approach ensures regulatory compliance while providing clinically meaningful insights. The convergence of different explainability methods on consistent biomarkers demonstrates robustness required for regulatory approval. Furthermore, counterfactual explanations provide actionable insights for clinicians, showing precisely which physiological changes indicate pain transitions.

The global surrogate models (decision trees with max_depth = 5) extracted interpretable rules that align with clinical knowledge, such as “IF ECG_RMS > 0.65 mV AND LF/HF_ratio > 2.5 THEN Pain_probability > 0.8.” Such rules can be directly integrated into clinical decision support systems, providing transparent reasoning that clinicians can verify against their experience.

### 4.4. Insights From Partial Dependence and Interaction Analysis

The PDP/ICE analysis provided crucial insights beyond traditional feature importance metrics, revealing the nature of relationships between ECG‐derived biomarkers and pain states. Three key findings emerged with important clinical implications.

*Complex nonlinear relationships*: The nonlinear patterns observed in features such as ecg_peak_to_peak (threshold effects) and ecg_skewness (peaked relationships) demonstrate that pain does not manifest as simple, proportional changes in physiological parameters. Instead, pain triggers complex, nonlinear modulations of cardiac function that may involve threshold effects, saturation phenomena, or optimal “signature” values. This complexity necessitates sophisticated analytical approaches such as machine learning rather than simple rule‐based detection algorithms.

*Feature interactions dominate marginal effects*: The apparent paradox of high permutation importance despite flat partial dependence curves for features such as rr_std and ecg_kurtosis reveals a critical insight: The most important biomarkers may derive their predictive power primarily through interactions with other features rather than independent marginal effects. This finding validates our ensemble learning approach and suggests that multivariate analysis is not merely beneficial but essential for reliable pain detection.

*Substantial interindividual variability*: The pronounced heterogeneity in ICE curves, particularly for ecg_skewness and ecg_peak_to_peak, indicates that different individuals exhibit distinct physiological “signatures” of pain. While the average population response provides useful insights, individual responses can deviate substantially. From a precision medicine perspective, this finding suggests that optimal pain assessment may benefit from personalized models that learn individual‐specific pain signatures through adaptive calibration.

These insights underscore the value of XAI techniques not only for building trust in automated systems but also for advancing our fundamental understanding of pain neurophysiology and its cardiovascular manifestations. The complex patterns revealed through PDP/ICE analysis would be invisible to traditional statistical approaches, highlighting the synergy between advanced machine learning and sophisticated interpretability methods in biomarker discovery and validation.

### 4.5. Addressing Healthcare Disparities

Objective biomarker‐based pain assessment could significantly reduce documented disparities in pain management [1, 3]. By relying on physiological signals rather than subjective interpretation, our system minimizes opportunities for conscious or unconscious bias. The model’s high specificity (87.56%) reduces false positives that might perpetuate undertreatment, while high sensitivity (75.23%) ensures pain is not missed in any demographic group.

However, we acknowledge that demonstrating actual disparity reduction requires prospective validation across diverse populations. The limited demographic information in the PMED prevents stratified analysis by age, sex, or ethnicity. Future studies must deliberately recruit diverse cohorts and perform subgroup analyses to confirm equitable performance.

### 4.6. Integration With Clinical Workflow

The practical implementation pathway distinguishes our approach from more complex alternatives. Integration requires only the following:

1. Access to standard ECG monitoring (already present in most clinical settings)
2. Computational capacity for real‐time random forest inference (minimal compared to deep learning)
3. Display interface for biomarker values and explanations

We envision a clinical dashboard displaying: (a) continuous pain probability with confidence intervals, (b) key contributing biomarkers highlighted, (c) alerts for significant changes, and (d) explanations accessible on demand. This design balances automation with clinical judgment, augmenting rather than replacing clinical assessment.

### 4.7. Limitations and Their Implications

Several limitations warrant careful consideration.

**FIGURE 11:**
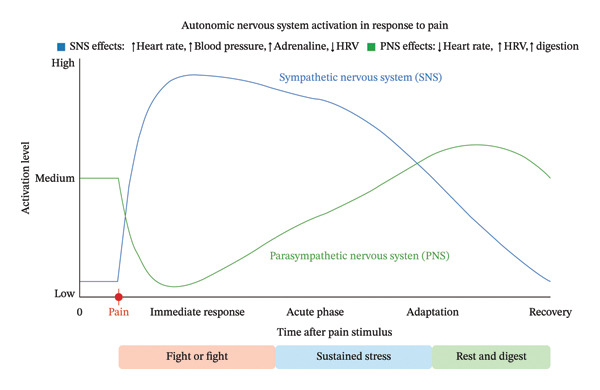
Diagram of the autonomic activation in response to pain.

*Experimental vs. Clinical Pain*: The PMED’s heat‐induced pain in healthy volunteers differs fundamentally from clinical pain. Heat pain primarily activates nociceptive pathways, while clinical pain often involves inflammatory, neuropathic, or mixed mechanisms. The autonomic responses we measured may not fully translate to chronic pain conditions where adaptation and sensitization alter physiological responses (see Figure 11).

*Limited Generalizability*: Our cohort’s restricted age range (18–65) and exclusion of comorbidities limits generalizability. Elderly patients, who experience the greatest pain management disparities, often have altered autonomic function and cardiac conditions affecting ECG parameters. Validation in these populations is essential.

*Single Pain Modality*: Exclusive reliance on ECG may miss pain types with minimal cardiac manifestations. Visceral pain, for instance, may trigger different autonomic patterns than somatic pain. Future iterations should investigate whether pain‐type–specific models improve accuracy.

*Threshold Effects*: The Bland–Altman analysis revealed systematic underestimation at high pain levels (> 70 on CoVAS scale) made from the patients themselves. This ceiling effect could result from autonomic saturation or reduced HRV at extreme sympathetic activation. Clinical implementation must account for this limitation, potentially using different models for severe pain.

*Temporal Dynamics*: Our analysis used fixed time windows, potentially missing rapid pain fluctuations or delayed autonomic responses. Real‐time implementation should explore adaptive windowing and temporal modeling techniques.

### 4.8. Future Directions and Research Priorities

Based on our findings and limitations, we propose the following research priorities.

*Clinical Validation Studies*: Prospective trials in surgical and chronic pain populations, comparing biomarker‐guided management to standard care. Primary outcomes should include pain control adequacy, opioid consumption, and patient satisfaction, with prespecified subgroup analyses by demographics.

*Personalized Model Development*: Leveraging the three phenotypes identified through ICE analysis, develop stratified models with phenotype‐specific biomarkers and thresholds. Machine learning techniques such as mixture‐of‐experts could automatically route patients to appropriate submodels.

*Multimodal Integration*: While maintaining ECG as the core modality, selectively incorporate additional readily available signals (respiratory rate from ECG‐derived respiration and blood pressure from arterial lines) to improve coverage of diverse pain types.

*Longitudinal Adaptation*: Develop online learning algorithms that adapt to individual patients over time, accounting for tolerance, sensitization, and changing baselines during extended monitoring.

*Clinical Decision Support Design*: User‐centered design studies with clinicians to optimize information presentation, balancing comprehensive biomarker data with cognitive load considerations. Implementation science methods should guide deployment strategies.

### 4.9. Implications for Pain Neuroscience

Beyond clinical applications, our XAI findings contribute to understanding pain mechanisms. The identified thresholds and nonlinear relationships suggest discrete state transitions in autonomic processing of pain, rather than continuous gradations. The heterogeneous response patterns indicate multiple pain processing phenotypes, possibly reflecting genetic, psychological, or experiential factors influencing pain perception.

The stronger importance of frequency‐domain HRV features (LF/HF ratio) versus time‐domain features for pain intensity estimation suggests that sympathovagal balance, rather than absolute autonomic activity, better reflects pain severity. This finding could inform development of targeted interventions modulating autonomic balance for pain management.

## 5. Threats to Validity

### 5.1. Internal Validity

- *Selection Bias*: Healthy volunteer sample may not represent clinical populations experiencing actual pain
- *Instrumentation*: ECG preprocessing and feature extraction choices could influence biomarker identification
- *Testing Effects*: Repeated pain stimuli might cause habituation or sensitization affecting later measurements

### 5.2. External Validity

- *Population Validity*: Results from young, healthy adults may not generalize to elderly or comorbid populations
- *Ecological Validity*: Laboratory‐induced heat pain differs from clinical pain contexts
- *Temporal Validity*: Cross‐sectional analysis may not capture longitudinal changes in pain responses

### 5.3. Construct Validity

- *Mono-operation Bias*: Relying solely on CoVAS for ground truth may not capture complete pain experience
- *Mono-method Bias*: ECG‐only approach might miss pain dimensions reflected in other physiological systems
- *Confounding Variables*: Anxiety, attention, and expectation could influence both pain ratings and autonomic responses

### 5.4. Statistical Conclusion Validity

- *Multiple Comparisons*: Extensive XAI analyses increase risk of Type I errors
- *Assumptions*: Random forest assumptions about feature independence may be violated with correlated ECG metrics
- *Sample Size*: While adequate for main effects, it may be underpowered for subgroup analyses

## 6. Limitations

This study presents several important limitations that warrant consideration. The experimental pain model employed, based on controlled heat stimulation in healthy volunteers, may not fully reflect the complex mechanisms underlying clinical pain states experienced by patients in real‐world healthcare settings. Additionally, the relatively homogeneous participant demographics in the PainMonit Experimental Dataset limit our ability to make robust generalizability claims about the model’s effectiveness in reducing disparities across diverse populations. The focus on a single pain modality, heat‐induced pain, raises questions about whether the identified ECG biomarkers would demonstrate a similar predictive value for other pain types, such as mechanical, chemical, or visceral pain. From a methodological perspective, the use of fixed time windows for feature extraction may fail to capture important temporal dynamics of pain responses that occur at different time scales, while ceiling effects observed at high pain intensities suggest potential limitations in discriminating between severe and extreme pain levels. Furthermore, the absence of an external validation dataset and the lack of comparison with clinical gold standards beyond self‐reported pain ratings represent gaps in establishing the robustness and clinical validity of our approach. Finally, although the computational requirements of our random forest–based approach are modest compared to deep learning alternatives, they may still present challenges for implementation in resource‐limited clinical settings where computing infrastructure is constrained.

## 7. Future Work

Building upon the foundation established in this study, several critical research directions emerge for advancing ECG‐based pain biomarkers toward clinical translation. The most immediate priority involves validating the identified biomarkers across diverse clinical pain populations, including postsurgical acute pain, chronic pain conditions, and cancer‐related pain, to establish their generalizability beyond experimental paradigms. Complementing this validation effort, randomized controlled trials directly comparing biomarker‐guided pain management protocols to standard care approaches are essential for demonstrating clinical utility and impact on patient outcomes. From a technical perspective, developing phenotype‐specific models that account for the distinct response patterns identified through our PDP/ICE analysis could enable more personalized pain assessment, while integrating complementary physiological modalities—such as blood pressure, respiratory rate, or limited EDA monitoring where available—may enhance accuracy while maintaining ECG signals as the core biomarker foundation. The implementation pathway should prioritize real‐time adaptive algorithms capable of continuous monitoring and dynamic recalibration, supported by carefully designed clinical decision support interfaces that present biomarker‐based insights in clinically actionable formats validated through user research with healthcare providers. Longitudinal studies investigating biomarker stability over extended monitoring periods and potential adaptation effects will be crucial for understanding long‐term reliability. Additionally, exploring advanced deep learning architectures that incorporate built‐in explainability mechanisms could potentially improve performance while maintaining the interpretability essential for clinical trust. Finally, establishing multisite research consortiums capable of generating large, demographically diverse validation datasets, and developing specialized pediatric models that account for developmental differences in pain physiology and autonomic regulation will be essential to ensure that these biomarker‐based approaches can equitably serve all patient populations requiring objective pain assessment.

## 8. Conclusion

This study demonstrates that ECG‐derived parameters can serve as reliable biomarkers for pain when analyzed through explainable machine learning techniques, providing objective, transparent pain assessment that could help address disparities in pain management. By focusing exclusively on ECG signals already collected in standard clinical care while implementing sophisticated feature extraction and dual‐model architecture, we achieved high accuracy in pain detection using signals that are routinely available throughout the perioperative period.

The dual‐model cascade approach—first classifying the presence of pain based on CoVAS ratings (0 vs > 0) and then estimating intensity for positive pain cases—provides a clinically intuitive framework that mirrors human decision‐making processes. This approach achieved 85.2% accuracy for binary pain classification and demonstrated good agreement between predicted and actual pain intensity values.

The explainability methods revealed important insights into which ECG features function most effectively as pain biomarkers, making the assessment process transparent and interpretable for clinicians. This transparency is essential for clinical adoption and could help address the biases that contribute to disparities in pain management.

From a precision medicine perspective, objective physiological biomarkers combined with XAI offer several advantages over traditional pain assessment methods. By reducing reliance on verbal self‐reporting and making the assessment process transparent, this approach could help ensure equitable pain management for all patients, including those from populations traditionally at risk for pain undertreatment.

While important challenges remain, particularly regarding translation of these biomarkers to clinical settings, this work establishes a foundation for more equitable pain assessment in everyday clinical practice. By identifying and validating ECG‐derived biomarkers for pain through machine learning approaches, we contribute to the growing field of precision pain management, where objective measures guide individualized treatment decisions.

## Funding

The funding for the doctoral programme is administered by Campus Bio‐Medico University of Rome through the CRUI‐CARE agreement, as Campus Bio‐Medico is the consortium lead and grant‐holding entity.

## Conflicts of Interest

The authors declare no conflicts of interest.

## Data Availability

The data that support the findings of this study are openly available in The PainMonit Database at https://figshare.com/articles/dataset/The_PainMonit_Database_An_Experimental_and_Clinical_Physiological_Signal_Dataset_for_Automated_Pain_Recognition/26965159.
